# Reddit Users' Experiences of Suicidal Thoughts During the COVID-19 Pandemic: A Qualitative Analysis of r/Covid19_support Posts

**DOI:** 10.3389/fpubh.2021.693153

**Published:** 2021-08-12

**Authors:** Allie Slemon, Corey McAuliffe, Trevor Goodyear, Liza McGuinness, Elizabeth Shaffer, Emily K. Jenkins

**Affiliations:** ^1^School of Nursing, University of British Columbia, Vancouver, BC, Canada; ^2^British Columbia Centre on Substance Use, Vancouver, BC, Canada; ^3^Indian Residential School History and Dialogue Centre, University of British Columbia, Vancouver, BC, Canada

**Keywords:** suicide, COVID-19, mental health, reddit, social media, qualitative research, self-disclosure

## Abstract

**Background:** The COVID-19 pandemic is having considerable impacts on population-level mental health, with research illustrating an increased prevalence in suicidal thoughts due to pandemic stressors. While the drivers of suicidal thoughts amid the pandemic are poorly understood, qualitative research holds great potential for expanding upon projections from pre-pandemic work and nuancing emerging epidemiological data. Despite calls for qualitative inquiry, there is a paucity of qualitative research examining experiences of suicidality related to COVID-19. The use of publicly available data from social media offers timely and pertinent information into ongoing pandemic-related mental health, including individual experiences of suicidal thoughts.

**Objective:** To examine how Reddit users within the *r/COVID19_support* community describe their experiences of suicidal thoughts amid the COVID-19 pandemic.

**Methods:** This study draws on online posts from within *r/COVID19_support* that describe users' suicidal thoughts during and related to the COVID-19 pandemic. Data were collected from creation of this subreddit on February 12, 2020 until December 31, 2020. A qualitative thematic analysis was conducted to generate themes reflecting users' experiences of suicidal thoughts.

**Results:** A total of 83 posts from 57 users were included in the analysis. Posts described a range of users' lived and living experiences of suicidal thoughts related to the pandemic, including deterioration in mental health and complex emotions associated with suicidal thinking. Reddit users situated their experiences of suicidal thoughts within various pandemic stressors: social isolation, employment and finances, virus exposure and COVID-19 illness, uncertain timeline of the pandemic, news and social media, pre-existing mental health conditions, and lack of access to mental health resources. Some users described individual coping strategies and supports used in attempt to manage suicidal thoughts, however these were recognized as insufficient for addressing the multilevel stressors of the pandemic.

**Conclusions:** Multiple and intersecting stressors have contributed to individuals' experiences of suicidal thoughts amid the COVID-19 pandemic, requiring thoughtful and complex public health responses. While ongoing challenges exist with self-disclosure of mental health challenges on social media, Reddit and other online platforms may offer a space for users to share suicidal thoughts and discuss potential coping strategies.

## Introduction

The coronavirus (COVID-19) pandemic has caused widespread societal disruption, with implications for nearly every aspect of daily life. There is growing recognition that the pandemic and associated public health restrictions are having adverse impacts on population-level mental health, with people across the globe struggling with greater levels of stress, worry, anxiety, and depression (1, 2). In Canada, for example, our recent cross-sectional monitoring survey with a nationally representative sample of adults identified significant deteriorations in mental health due to the pandemic, including widespread experiences of poor coping, self-harm, and suicidal thoughts (3). Findings of this nature are mirrored in other national contexts, such as in China (4), Japan (5), Italy (6), the United States (7), and the United Kingdom (8), and are also substantiated through several international systematic reviews (2, 9, 10).

Increasingly, it is suggested that the psychosocial stress of the pandemic may contribute – or is already contributing – to high and rising rates of suicide attempts and deaths (11–14). While empirical evidence regarding the association between COVID-19 and suicide mortality is limited, and considered a lagging indicator not expected to reflect actual numbers for years afterwards (15), findings from a recent systematic review of past epidemics (e.g., SARS, Ebola) support a link between virus outbreaks and suicide-related outcomes, including attempts and deaths (16). There is also a growing literature demonstrating an association between the COVID-19 pandemic and suicidal *thoughts* (2, 7, 17). For example, while in 2016, 2.5% of the general population in Canada reported suicidal thoughts in the past-year (18), the prevalence of suicidal thoughts was identified as 6% over a two week period in May 2020 (3) and 10% over a two-week period in September 2020 (19). Having suicidal thoughts, regardless of whether or not they are acted upon, is a clinically significant indicator of poor mental health and is thus, in and of itself, cause for concern (20). Investigation into the experience of suicidal thoughts amid the pandemic has tended to be epidemiological in nature, focused on describing the magnitude of suicidal thinking at a population level, with far less attention directed toward the lived and living experience of suicidality. Qualitative research approaches can work in complement with epidemiological methods to address these shortcomings (21), as qualitative methods are well suited to characterizing – in depth – the ways in which COVID-19 and related health sequelae, including suicidality, are experienced, understood, and ascribed meaning. To date, however, there is a paucity of qualitative research on suicidal ideation in the COVID-19 context. Notwithstanding this gap, the evidence base signals a need for concerted efforts to curb foreseeable surges in suicidal ideation and related mental health morbidity amid the pandemic (5, 11, 22).

Tailored strategies are needed to describe and, ultimately, respond to the rising prevalence of suicidal thoughts amid the COVID-19 pandemic. Of critical importance here is investigation into the specific experiences through which the mental health consequences of the pandemic are produced. Indeed, the pandemic has created unprecedented challenges for people and communities in their day-to-day lives, social and family relationships, and broader structural environments. It has been postulated that noteworthy determinants and “stressors” related to suicidality could include perceptions of fear and unease caused by the threat of the virus, experiences of loneliness and social isolation, restricted opportunities for meaningful connection due to physical distancing guidelines, quarantine, loss of employment and financial instability, and reduced access to mental health and other healthcare services, among other challenges (5, 11, 12, 22, 23). At this juncture in the pandemic, international research into the drivers and contexts of suicidal thinking has largely been limited to hypotheses and associated calls to action (11, 24, 25). Even if informed by (pre-pandemic) evidence, this speculation is insufficient for understanding determinants of rising suicidality over the course of this ongoing public health crisis – especially given its scale. Qualitative research holds great potential for delineating and nuancing these determinants, as the open-ended nature of qualitative inquiry centers the “*how*” and “*why*” instead of just the “*what*”, creating spaces for individuals and communities to articulate their own viewpoints, meanings, and experiences (21). It is for these reasons and others, as detailed in a recent systematic review by Johnson and Vindrola-Padros (26), that qualitative approaches have distinct utility in investigating context-specific issues, such as those stemming from the COVID-19 pandemic, and to inform public policy responses to complex health emergencies.

With complex health crises, there will undoubtedly be evidence gaps and corresponding challenges in developing appropriate policy responses. Among emerging public health priorities, there is demand for research to address existing gaps in knowledge and practice related to suicide prevention in the context of COVID-19. To do so comprehensively will require targeted methodological approaches, including, for example, focused qualitative studies, which can pragmatically and richly characterize how features of the pandemic context may influence individual experiences of suicidality. Here, pertinent and potentially fruitful sources of qualitative data include public commentary posted to online forums, as these virtual platforms can capture and display health experiences in “real time” through mediums that are readily available to the public and (albeit inadvertently) to researchers (27). One such platform, Reddit, is among the most visited websites in the world and brings together users from more than 200 countries (28, 29). It is a social media platform where users create special forums, called “subreddits”, which are utilized as virtual spaces for social gathering and discussion related to community-identified topics or issues (29). The nearly global reach of Reddit and its semi-anonymous design (30) make it well suited for discussions of – and related research explorations into – geographically far-reaching issues and stigmatized topics, including mental health and suicidality as related to COVID-19. Indeed, research from prior to the pandemic has already demonstrated – both qualitatively and quantitatively – the transformative ways in which online discussion forums create open, supportive, and widely accessible spaces for peer-to-peer exchange of dialogue and information related to mental health and suicidality (29, 31–34).

Applied to the COVID-19 pandemic, one mathematical modelling study using Reddit data recently demonstrated a statistically significant spike in forum posts related to suicidality, health anxiety, and associated stressors (e.g., economic stress, isolation) in conjunction with the start of the pandemic (27). The authors of this study identified a need for research to elucidate deeper understandings of the nature of individual posts and collective forums pertaining to suicidal ideation, including, in particular, the forum *r/Covid19_support*, which contains a large portion of the study's identified suicide-related posts (27). To our knowledge, however, the use of Reddit data to qualitatively understand and nuance experiences of suicidal ideation in the context of the pandemic remains underexplored. The present study is responsive to this research gap and, more generally, contributes to addressing the paucity of research exploring suicidal thoughts within the evolving pandemic. Specifically, the objective of this study is to examine online posts expressing suicidal thoughts within the Reddit *r/COVID19_support* community, with an intent to explore how users describe these experiences of suicidal thoughts as related to COVID-19 pandemic. The overarching aim of this analysis is to provide current data for informing public health efforts to promote mental health and prevent suicide as the pandemic continues to unfold.

## Materials and Methods

This study seeks to address the research question: How do Reddit users within the *r/COVID19_support* community describe their experiences of suicidal thoughts amid the COVID-19 pandemic? Our interdisciplinary research team includes social sciences researchers, health researchers, and mental health clinicians. We approach this analysis through a social constructivist lens, thus understanding Reddit users' experiences of suicidal thoughts as being uniquely situated within individual and collective experiences related to the COVID-19 pandemic. Further, as a social constructivist philosophical positioning contends, “meanings are constructed by human beings as they engage with the world they are interpreting” (35). Therefore, we recognize that this analysis is co-constructed through our research team's immersive and collaborative engagement with the study data among the research team. Rather than present generalizable and objective ‘truths' of the experience of suicidal thoughts amid the pandemic, this analysis seeks to present nuanced descriptions of suicidal thoughts within one community of Reddit users.

### Study Context

Data from this study are drawn from user posts on the online Reddit *r/COVID19_support* community. This subreddit was founded February 12, 2020 in response to the increasing spread of the COVID-19 virus. The Community Description of *r/COVID19_support* characterizes the subreddit as an online space that “offers help and support to those feeling overwhelmed by the COVID19 pandemic. It [is] a place to share advice, coping mechanisms and to feel calm and supported” (36). The subreddit is further governed by Community Rules and Guidelines, which include “Offer support, not opinions” and affirmation that “This is a safe space”. Multiple active moderators are involved in monitoring posts and comments, with the Community Description explicitly forbidding content that discusses “policies or politics relating to lockdown restrictions.mask wearing or other politicised issues,” or that does “not offer supportive advice, understanding and kindness” (36). Moderators are Reddit users, some of whom choose to remain anonymous, and others who self-identify as academics, health experts, or clinicians. As of December 31, 2020, this subreddit had approximately 33,300 community members, making it one of the top COVID-19 related communities on the Reddit platform. This subreddit is distinct from other COVID-19 related subreddits in that it is a space for users to seek help and support, while other communities tend to have more epidemiological and research-intensive aims, such as to support “scientific discussion of this global public health threat” (*r/COVID19*), or to “monitor the spread of the disease COVID-19” (*r/Coronavirus*).

While *r/COVID19_support* Rules and Guidelines state that the forum offers “non-judgmental peer support”, they do not explicitly address issues of users expressing mental health challenges or suicidal thoughts. However, in March 2020, Reddit partnered with the organization Crisis Text Line to provide Reddit users in crisis with live text support. As a new feature of this partnership, any Reddit user can ‘flag' posts that express suicidal thoughts, and Reddit will send an immediate private message to the poster providing various resources, including information to directly contact a crisis counsellor via text. As this occurs through private, direct messaging, there is no publicly available data indicating the frequency with which this flagging system has been used or which posts may have been flagged.

### Data Collection

Data were gathered from the point of the subreddit creation on February 12, 2020, through to December 31, 2020. To collect Reddit posts within *r/COVID19_support* related to suicidal thoughts, we conducted a search of the subreddit using the term “suicide,” which includes variations of the term (e.g., “suicidal”). Reddit's search function captures search terms located within the original post and within any subsequent comments. Testing of search terms such as “kill myself” did not lead to the capture of additional posts; therefore, further terms were not used. Search results were sorted using the “New” function to chronologically sort all posts. Posts were included if the original poster identified experiencing suicidal thoughts, feelings, or behaviours during the COVID-19 pandemic. Posts were excluded if the original poster only referenced suicidal thoughts prior to COVID-19 or if they expressed mental health challenges without explicit mention of suicidal thoughts. Each post and associated comments were captured directly from Reddit using the NCapture add-on tool for internet browsers, hosted by the qualitative data analysis software NVivo. Data were uploaded to NVivo Version 12 (37), which was used to facilitate data analysis. All data were analyzed as written in the original posts, and quotations in this paper are presented verbatim, though spelling mistakes have been corrected for readability.

### Data Analysis

The primary data analyzed within this qualitative inquiry were original posts from Reddit users; however, original posters' responses to comments were found to often provide additional detail and nuance, extending narratives from the original post, and were also included in this analysis. The analysis of Reddit posts identifying users' experiences of suicidal thoughts and the associated comments was guided by Braun and Clarke's (38, 39) thematic analysis. Firstly, posts were read line-by-line in an iterative process and salient trends in the data were discussed among the research team. Next, open coding was conducted among the research team to broadly develop codes reflecting users' narratives of experiencing suicidal thoughts. Rather than creating a ‘fixed' set of codes in advance of the data analysis process, open coding involves the creation and evolution of codes throughout the analysis process, including renaming/redefining, ‘splitting', and combining codes (40). In this analysis, open coding unfolded throughout the iterative data analysis process, and involved ongoing discussion among the research team regarding how codes were applied to the dataset. The codes generated through this process were then examined to develop themes that represented Reddit users' experiences of suicidal thoughts amid the COVID-19 pandemic. The development of themes occurred through discussion among the research team to identify key patterns within and between codes. To ensure that findings from this study are credible and reflect integrity throughout the research process, the research team adhered to guidelines for ensuring rigor within qualitative inquiry (41).

### Ethics

All Reddit posts included in this analysis are publicly available data. Ethical approval was therefore not required for this study, which was confirmed in the authors' correspondence with the University of British Columbia Behavioural Research Ethics Board. However, analysis of Reddit and other social media data is an emerging area of inquiry, and both researchers and institutional review boards have grappled with ethical engagement with these forms of public data, including possible strategies for mitigating risks related to confidentiality and consent (42, 43). Given the personal and sensitive nature of the posts and comments and following from other researchers' use of Reddit posts as a qualitative data source for understanding mental health experiences (29, 34), we do not report specific dates of posts and we have replaced all usernames with numerical identifiers.

## Results

A total of 83 posts from 57 distinct usernames were included in this analysis. Fifty-one users had only one post included in analysis, while the remaining six users had between two and 14 posts. Posts were dated from March 10, 2020 to December 25, 2020. There were 1,992 total comments (averaging 24 comments per post). While comments from other Reddit users were not included in this analysis, responses to comments written by the original posters were included in this dataset, as they provided additional nuance and detail to the experience of suicidal thoughts. As Reddit does not collect personal information about users, we are unable to offer demographic characteristics of the sample; however, some posts self-identified personal attributes. For example, the range of ages appears to include teenagers who describe high school experiences to middle-aged people who reference older children. Most users did not mention their location, though some users indicated living in countries including Canada, the United States, Brazil, and Russia.

Thematic analysis of original posts and posters' subsequent responses to comments yielded three broad themes that characterized *r/COVID19_support* community members' experiences of suicidal thoughts. Firstly, posts described the lived and living experience of suicidal thoughts during the COVID-19 pandemic. Secondly, users situated their experiences of suicidal thoughts within multiple and various pandemic stressors. Lastly, original posts and posters' responses to comments discussed managing suicidal thoughts, through coping and seeking support, while also identifying ongoing challenges. Each of these themes is presented in detail below.

### Lived and Living Experiences of Suicidal Thoughts During the COVID-19 Pandemic

Across posts, Reddit users described deteriorations in mental health and experiences of suicidal thoughts related to the COVID-19 pandemic. For example, one user described an “absolute feeling of hopelessness regarding COVID in general” *(OP18)*, while another stated, “my mental health has gone to absolute shit since the pandemic started…I battle suicidal ideation nearly every day” *(OP35)*. Many posts described ongoing suicidal thoughts persisting over the course of the pandemic: as shared by one young adult, “every time I wake up now I wish someone would kill me…Every time I think of death I feel relieved” *(OP36)*. For many, the pandemic was described as having a cumulative effect, with mental health and suicidal thoughts worsening over time. One individual recounted “experiencing a growing sense of dread and a lot of suicidal thoughts and emotions” *(OP30)*, while another user wrote

“Those first three months of quarantine I held it together and was doing pretty damn well. In June things started to slip, and this month I've lost it…I'm on the brink of either a mental break or suicide about now. (OP47)”

For others, suicidal thoughts grew in intensity at particular points of the pandemic, such as periods of lockdown. One user described having had frequent and serious suicidal thoughts during a prior lockdown in their region and expressed apprehension that this would reoccur with subsequent lockdowns: “so, when the second lockdown is announced, i guess i'll have to kill myself because i have no more reasons to keep living and nothing to wait for in the future” *(OP19)*.

While many posts recounted ongoing experiences of suicidal thoughts, others appeared to be written during moments of considerable distress. One user described learning of a co-worker's death from COVID-19 and stated, “now I'm panicking again…there's too much pain and I'm constantly dwelling on suicide just to get out” *(OP32)*. Another Reddit user's post called for support while experiencing a crisis of “intrusive suicidal thoughts” – “Please help me if you can. I'm not doing great…I'm absolutely bawling my eyes out just typing this” *(OP55)*. Many posts similarly described suicidal thoughts as being associated with complex emotions and emotional patterns, including “rumination”, “anxiety”, “depression”, and feeling “angry”, “scared”, “helpless”, and “completely out of control”. Many users identified feeling “hopeless”, with “nothing to look forward to”. While most posters described primarily having suicidal *thoughts*, others expressed concern that their thoughts may progress to actions and, potentially, death. As one person stated, “I'm legitimately suicidal” *(OP29)*, while another user reported, “I genuinely think my life is about to end” *(OP41)*.

### Situating Experiences of Suicidal Thoughts Within Pandemic Stressors

Across all posts, users situated their experiences of suicidal thoughts within the context of specific stressors related to the COVID-19 pandemic, reflecting on the inter-relationship between pandemic conditions and their own deterioration in mental health and emergence of suicidal thoughts. With representative quotes from Reddit posts, Table 1 presents seven prominent stressors, discussed in detail below: social isolation; employment and finances; virus exposure and COVID-19 illness; uncertain timeline of the pandemic; news and social media; pre-existing mental health conditions; and lack of access to mental health resources.

**Table 1:** Pandemic stressors shaping *r/COVID19_support* Reddit users' experiences of suicidal thoughts.

**Stressors**	**Representative quotes**
Social isolation	“I feel completely worthless and like I have no purpose at all. I need people.I need my people. Phone calls and computer screens aren't enough.they feel shallow and empty and hollow.” *OP42* “And I've spent almost 60 days without going past the walls of my house. Talking to my friends through calls isn't enjoyable and distracting anymore. It feels like they are on other planet. It feels like no one cares about me at all… I have nothing. I have no one to talk to. Feels like I'm reliving the same fucking day over and over. Feels like time doesn't exist anymore.” *OP34* “Covid has just dominated all of our lives and there's no end in sight. It's just seems like its endless and it's killing me. The social distancing and online school is worst of all… Meeting people online just isn't the same it doesn't feel real for some reason. I'm so lonely it hurts every night it's like slow excruciating torture from my own mind. It so bad I want to die.” *OP36* “I have diagnosed depression and am on the autism spectrum. As a result the little social interaction I do have is incredibly important and bordering on life saving to me… people are saying ‘don't worry, you can hang out with your friends virtually!' which is not much help if you don't have them already. The thought of being forced to not leave the house is devastating.” *OP26* “I'm so sick of feeling like every single action I take has this enormous moral weight to it that it didn't used to have. Want to hang out with a friend? Cool, if I have it and I'm asymptomatic I could potentially kill her at-risk family.” *OP08*
Employment & finances	“I'm scared to go into work, but if I don't then I'm a missed paycheck away from homelessness again…there doesn't seem to be any hope and there's too much pain and I'm constantly dwelling on suicide just to get out.” *OP32* “not having a job feels like the society doesn't need you and can go well or even better off without you, which is the reason why the job loss pulled the trigger so far i actually came up with the suicide plan that will work 100%” *OP19* “Right now I don't think surviving the pandemic is worth it… My finances are permanently destroyed from covid.” *OP31*
Virus exposure and COVID-19 illness	“I am paranoid someone else will bring it into the [workplace] and I'll get it or bring it home to my mom and dad or unknowingly give it to my boyfriend… I don't want anything bad to happen to him. He's 25 and works in [a front line essential worker position].” *OP12* “I just need to get over this absolute feeling of hopelessness regarding COVID in general. It's so easy right now to go ‘what's the point, I'm just going to get COVID and die.”' *OP18* “I limit myself to 1 trip out of the room per week to get groceries and for less than an hour since I'm still not sure what risk I am at due to my heart…I feel hopeless and afraid.” *OP48* “I moved back with my mother last year, she's 68 and does have a lot of health problems…Because I live with her, I haven't gone out at all in the past 2 months and a half…I'm trapped in an already bad life, this situation has taken my hope and my outs away from me.” *OP17* “The worst-case scenario has been incredibly overwhelming in my head, and I've been having suicidal thoughts entering my thoughts… I can't imagine continuing forward, at the age of 20 in my college years, and dealing with the loss of a parent or sibling in the process…I don't know what can help me. I'm just heartbroken that I'm even thinking about an early ending if things go that way…” *OP55*
Uncertain timeline of the pandemic	“There does not seem to be an end date to it… And I honestly don't think it is catastrophizing. This is an actual catastrophe, and if anything events have played out worse than I anticipated.” *OP26* “Right now it's looking more and more like 2021 will get canceled as well, and essentially suicide is now my best option because it means things like online school and work from home will become permanent features of society.” *OP31* “I posted like a week ago asking if this ‘new normal' was the permanent normal (because it really feels that way). I really appreciate all of the comments reassuring me that there was hope for the future. But it still really doesn't feel that way and this is really taking a toll on my mental health. I have seen a few articles recently by experts…that this (social distancing) is going to have to be around for years if not forever. This is apparently even if we get a vaccine.” *OP49*
News and social media	“Pfizer's recent announcement about how the US won't get more vaccines until July has me devastated. I've struggled with suicidal ideation throughout much of this pandemic and have been looking to the vaccines as a ‘light at the end of the tunnel' for the past month.” *OP02* “After I read the mink mutation news I lost all hope of having a happy future.” *OP25* “I have been kind of lurking in r/Coronavirus for some time, but I know I should probably stop. The majority of my suicidal episodes are triggered by something I read there, and I constantly get trapped in doomscrolling.” *OP34* “I'm so unsure and beyond freaked out by all the news, panic, and speculation from everyone from news anchors to doctors to people on other subs and on Facebook pretending they're experts.” *OP11* “I am in my early 30s and my spouse is in her later 30s. We both have hypertension. I just read on r/coronavirus that a doc from China has said that people with hypertension have a 50x higher chance of dying! And I just finished an article by NatGeo is pretty much saying the same thing…” *OP15*
Exacerbation of pre-existing mental health challenges	“I have a history of depression and anxiety, as well as three suicide attempts. This virus is making me seriously consider attempt number 4, especially when I see that the worst case scenarios are likely to occur.” *OP33* “So this past year I've been in recovery from severe suicidal ideation, finally reaching a point where I wanted to live more often than not about four months ago. All of my progress can be attributed to fighting hard to make necessary changes in my life. Almost ALL of those tangible changes have now been undone because of covid and lockdowns.” *OP46* “I keep beating myself up in my head that I didn't take steps to resolve my issues with depression/anxiety sooner, so I was in a better living situation/support network during the pandemic.” *OP24*
Lack of access to mental health resources	“I've tried speaking to an online therapist about my issues and it's not really working. Online socialization just doesn't really work for me.” *OP31* “My suicidal thoughts have become intrusive and compulsory…I found out my teletherapy/psychiatry copays were being waived due to the pandemic…and will be billed at $60. That puts me at $540 through next week. I can't afford this.” *OP06* [In response to a comment suggesting seeking emergency mental health care]: “i'm afraid of institutionalization and of getting exposed to the virus if i go there” *OP46*

Firstly, social isolation was consistently recognized as a key stressor contributing to suicidal thoughts. Users described loss of regular social contact and connectedness, including from gatherings with friends and family members. Virtual contact, including phone calls and online contact, tended to be described as “not the same” and insufficient for addressing the profound loneliness and isolation related to lockdowns and other pandemic-related restrictions. While many described missing friends and family, others also identified an inability to meet new people with loss of in-person opportunities for work, school, and social gatherings. For many, this loss of social contact was exacerbated by challenges of living alone or with family members with whom they have strained relationships. The mental health impacts of social isolation led to some individuals questioning whether they could sustain adherence to public health restrictions. As stated in one post, “I know this is going to be controversial, but come June 2021 and I'm not giving a crap anymore about this social distancing shit… it's either this or suicide” *(OP25)*. However, decisions to seek out in-person contact to cope with suicidal thoughts were described as holding “moral weight”, as gatherings might lead to virus transmission. This tension between the risks and mental health benefits of social contact was itself a stressor, further contributing to suicidal thoughts.

Additionally, employment and finances were identified as considerable stressors during the pandemic. Many posts described ongoing financial insecurity related to job loss and reduced income, as well as concerns about the unavailability of government benefits. Further, individuals who reported being currently unemployed – including from recent job loss or student status – expressed fears that future job prospects would be limited. While many users reported having stable jobs, the pandemic introduced new work-related stressors. For example, while some users described being anxious about potential exposure to COVID-19 in the workplace, they also indicated that they could not take time off or a leave from their positions, as they would not be able to cope with the lost income. Conversely, working from home was identified as difficult, isolating, and unsustainable, with one user anticipating the need to change careers if working from home was to become permanent.

Many posts identified stressors related to the pandemic itself, including exposure to COVID-19, uncertain duration of the pandemic, and news coverage regarding the pandemic. Virus exposure and the potential for serious illness was a frequent stressor, with posters expressing fear for themselves and for family and friends, particularly related to underlying health conditions. Many reported fears of potential COVID-19 infection leading to severe symptoms, hospitalization, and death, and described suicide as a means to avoid these outcomes – particularly, with respect to keeping their loved ones healthy and safe. Additionally, posts expressed users' feelings of hopelessness and distress related to the uncertain timeline of the pandemic. Many individuals wrote of concerns about having to endure indefinite public health restrictions such as lockdowns and physical distancing mandates, with fears that the pandemic may persist for years. These concerns were compounded by news coverage from both official news media sources and social media. Rather than help individuals gain information about the pandemic, reading news updates about COVID-19 was predominantly described as a contributor to poor mental health and suicidal thoughts. For example, posts described users' experiences of having suicidal thoughts after reading news stories about new research showing elevated risks for illness among those with certain underlying health conditions, or expert opinions on the barriers to returning to ‘normal life.' Social media, including Reddit itself, was also occasionally identified as a source of stress, with users describing having suicidal thoughts “triggered” after reading untrue, “dubious”, and “fear-mongering” posts.

In situating their experiences of suicidal thoughts within the pandemic context, many users identified having pre-existing mental health challenges, including depression, anxiety, obsessive compulsive disorder, and histories of trauma and post-traumatic stress disorder. Additionally, some posts described a history of suicidal thoughts or past suicide attempts. Almost all users who identified having pre-existing mental health challenges reported that their mental health had deteriorated substantially during the pandemic and due to corresponding stressors, such as social isolation and fear of illness. For many, intersecting pandemic-related stressors were described as “undoing” previous gains made toward mental health and well-being and exacerbating prior mental health challenges and suicidal thoughts. Further, both those who identified a history of mental health challenges, and those who did not, reported worsening access to mental health resources. Some of these accessibility issues stemmed from challenges with mental health care systems prior to the pandemic, including high costs associated with mental health supports, such as counselling or therapy. Other issues of mental health resource accessibility were directly related to the COVID-19 pandemic, including a shift from in-person to virtual mental health supports, which were identified by some as less helpful. Additionally, two individuals reported concerns about the potential for virus exposure within institutionalized mental health settings, preventing both from presenting to hospital for support despite having serious suicidal thoughts.

### Managing Suicidal Thoughts: Coping, Seeking Support, and Identifying Ongoing Challenges

The posts included in this analysis reflect users' experiences of suicidal thoughts in the context of considerable stressors related to the COVID-19 pandemic, and as such, predominantly illustrate mental health challenges and experiences of distress. However, many posts described various strategies for coping and approaches to seeking support to manage suicidal thoughts, which are described below.

#### Coping

Across posts, users expressed an *inability* to cope with stressors related to the pandemic, which in turn contributed to experiencing suicidal thoughts. However, some users described strategies undertaken in attempt to cope with these suicidal thoughts, predominantly through engaging in hobbies and activities that were still possible despite pandemic restrictions. These hobbies were described as important means of coping with suicidal thoughts, and in otherwise staying healthy and well. For example, some users described taking up new hobbies that could be done at home, while another individual stated, “I discovered cleaning and disinfecting the house really helped me ease down a lot” *(OP43)*. Others identified that outdoor activities with lower risk of virus transmission were helpful for managing suicidal thoughts. As described by one user

“The last few months I've been going for walks and runs outside, and meeting up with a few friends occasionally (always 6+ feet apart, with masks on the entire time, and outdoors) to do things like hiking and walking. Both the exercise/outdoor time and getting to see people has made a HUGE difference in whether I could even imagine surviving the rest of this… (OP01)”

#### Seeking Support

Despite new and pre-existing barriers to mental health care, users indicated that mental health supports and treatment were an important coping strategy for managing suicidal thoughts. Many individuals described taking prescribed medication for underlying mental health challenges and engaging in regular therapy or counselling. These strategies were reported to be helpful for managing suicidal thoughts, with one individual detailing, “my suicidal thoughts have gone from omnipresent to brief and occasional” *(OP34)*. Suicide hotlines were also identified by some as helpful resources, with one user reporting having called a local suicide hotline, and another stating they had the phone numbers of these resources on hand “in case I need to talk to somebody” *(OP42)*.

Beyond seeking formal mental health support, for *r/COVID19_support* subreddit users, posting online about their experiences of suicidal thoughts was identified as a means to share one's story and seek support from others. Writing about and sharing experiences was reported to be cathartic, with one user stating, “i needed to get this off my chest” *(OP54)*. Another individual described how posting online about their experiences contributed to better identifying their own strengths: “Typing this all out makes me realize how much I'm currently going through and I'm impressed I can still otherwise keep it together on the outside” *(OP35)*.

#### Identifying Ongoing Challenges

Although some coping strategies used to manage suicidal thoughts were described as helpful, overall, coping strategies and supports were identified as insufficient for managing suicidal thoughts amid the considerable stressors of the pandemic. Coping strategies that supported management of mental health challenges prior to the pandemic were identified as challenging or impossible within the pandemic context. For example, users indicated that they could no longer seek in-person connection with friends and family, engage with in-person mental health supports, participate in certain group recreational activities, and travel, including to see loved ones living abroad. As described in one post

“In the past when I'm mentally pretty rough I've always gotten through by doing things I love like eating at my favorite spots, going shopping, going to the gym, and just going out and being around people so I'm not isolated in my house. Now I don't even have the options to do that and I'm really worried. (OP28)”

Other users stated that coping strategies that were possible during COVID-19 helped only “temporarily” and were “getting less and less effective due to things getting more and more stressful” *(OP31)*. As described by one individual, “therapy and meds are useless when you have real reasons to end your life” *(OP35)*. The pandemic was consistently articulated as an unprecedented stressor, contributing to serious suicidal thoughts that were not easily managed by previously successful coping strategies.

## Discussion

The COVID-19 pandemic has had considerable impacts on mental health, including increased prevalence of depression, anxiety, and other mental health conditions, as well as self-reported deteriorations in mental well-being (1, 3, 44). Relatedly, researchers and public health officials have raised concerns that suicide rates may increase in the context of the various challenges of the pandemic, citing widespread unemployment, social isolation, fear, and barriers to mental health treatment as potential contributors (11, 13, 45, 46). Khan and colleagues (14) note that while suicide is a complex and multifactorial phenomenon, the COVID-19 pandemic has created a social context that introduces and exacerbates a variety of stressors, which have contributed to attempted and completed suicides among some individuals. Population-level evidence of increasing suicide rates remains limited; however, emerging research illustrates that the social context of the pandemic has contributed to growing rates of suicidal thoughts (3, 7, 47). While some research has begun to quantitatively examine associations between suicidal thoughts and socio-demographic characteristics or COVID-19 related conditions (17, 23), there is a dearth of qualitative research supporting more nuanced understandings of the experience of suicidal thinking, in particular, individuals' perspectives on contributors to suicidal thoughts in the pandemic context. As a semi-anonymous forum where users can share personal stories and receive support, Reddit data presents a unique opportunity to explore how members of an active online community are experiencing suicidal thoughts related to the pandemic, and to gain rich insights into the ways in which the pandemic context variously contributes to these experiences. This paper presents data from 83 posts within the Reddit community *r/COVID19_support* that detail users' suicidal thoughts. These data contribute to addressing gaps in qualitative inquiry into the mental health impacts of the COVID-19 pandemic, particularly related to experiences of suicidal thoughts among individuals that have access and comfort using online forums and resources. Data from this thematic analysis illustrates that among Reddit users, people are having suicidal thoughts related to pandemic conditions, corroborating and nuancing existing quantitative data on this priority issue among the general population (3, 47–49).

In *r/COVID19_support* posts included in this analysis, Reddit users reported multiple pandemic-related stressors that they identified as contributing to their experiences of suicidal thoughts. Specifically, concerns related to employment and finances were commonly referenced in users' posts, which has been consistently identified as a potential contributor to increases in suicidal thoughts and behaviours amid widespread unemployment (11, 45, 50–52). Concerningly, this study also documented Reddit users' perceptions about how their pre-existing mental health challenges – including suicidal thinking – are being exacerbated in the pandemic context, with previous ‘work' toward addressing mental health challenges and suicidal thoughts potentially being ‘undone' by pandemic-related stressors. These findings align with literature from prior to the pandemic demonstrating that having a pre-existing mental health condition is a strong predictor for suicide (53–55), as well as more recent scholarship identifying baseline (i.e., pre-pandemic) mental ill health as a contributor to poor(er) mental health within the context of COVID-19 (3, 56, 57). More generally, findings from this study paint a complex picture illustrating associations between Reddit users' suicidal thoughts and various stressors amid the COVID-19 pandemic, suggesting that there are multiple and intersecting contributors to this experience. These include complex and compounding pandemic-related stressors and, in many cases, prior stressors such as underlying mental health, social, and financial challenges. Insights into evolving “drivers” of suicidality presented in this analysis suggest that creative and multi-pronged supports may have distinct potential for most comprehensively responding to suicidal thinking during and beyond the pandemic – among members of the Reddit community and perhaps more broadly. There remains demand, however, for additional research and practice efforts to identify and develop supports that can optimally promote the mental health of Reddit and other online resource users, and to tailor these supports in ways that are responsive to distinct community needs and experiences.

Social isolation was consistently identified as a considerable contributor to suicidal thoughts in this study, with users' posts detailing feelings of loneliness, lack of social support, and difficulty forming and maintaining relationships. While the associations between loneliness and suicidal thoughts are well-documented outside of the pandemic context (58, 59), the COVID-19 pandemic presents uniquely challenging circumstances, with widespread isolation from physical distancing mandates, individual periods of quarantine, and regional lockdowns. Social isolation has been projected to contribute to increases in suicide rates amid the pandemic; however, the relationship between social isolation and suicidal thoughts in this context remains underexamined (13, 60). Findings from this analysis illustrate that online social interaction – despite its widespread promotion through public health messaging – may be insufficient in protecting against loneliness, disconnection, and isolation as contributors to suicidal thoughts. Further, posts from Reddit users describe a complex grappling with the ‘moral weight' of balancing in-person socializing as a positive contributor to mental well-being, and the potential consequences of virus transmission associated with any decision to connect in-person with others. Adherence to public health mandates is undoubtedly crucial for reducing virus transmission; however, the mental health impacts of these restrictions is an ongoing challenge (61, 62). Thus, drawing on established public health ethics principles of reciprocity and least restrictive means (63), increases in pandemic-related public health restrictions should be accompanied by reciprocal, compensatory efforts to safeguard population-level mental health. For example, alongside public health messaging to curb virus transmission through restricting activities and physical contacts, there is also a need to support mental well-being through additional messaging about safe options for connecting with others virtually and in-person, where local guidance allows – for example, taking a physically distanced walk with others. Such messages may help provide options for individuals – such as the Reddit users represented in this paper – struggling to comply with public health mandates to mitigate some of the suicidal thinking resulting from sustained restrictions impacting social connection (64–66). The implementation and evaluation of this sort of public messaging is also likely to benefit from co-design with people with lived and living experience of mental health challenges, including suicidal thinking, as drawing from their expertise is central to enhancing the appropriateness and impact of suicide prevention efforts (67).

Data from this study represents posts from only one of many Reddit communities related to mental health, suicide, and the COVID-19 pandemic. Yet, it includes numerous posts from individuals experiencing suicidal thoughts during the pandemic and captures the early days of the spread of COVID-19 through to the end of 2020. Across posts, users shared personal information about their experiences, with many asking explicitly for help and support and indicating that the process of posting online was cathartic and contributed to coping with mental health challenges. Other analyses of mental health self-disclosure and help-seeking on Reddit outside of the pandemic context similarly suggest that this online platform has become a space for many individuals to discuss their personal mental health struggles and gain support and advice from others (34, 68). Semi-anonymous platforms such as Reddit may serve as one meaningful outlet for connecting with others experiencing similar mental health challenges, particularly given the considerable stigma associated with mental ill health in society (30). Zhen and colleagues (69) further demonstrate that in the pandemic context, young adults' self-disclosure on social media contributed to reduced stress, suggesting that engaging with and supporting others online may protect against the mental health challenges associated with the pandemic. However, formal mental health responses typically focus on “official” virtual mental health resources, such as apps and online programs, which to date, have had low uptake and lack of sustained use (70, 71). While it remains crucial to strengthen publicly available mental health supports and treatment options (72), findings from this analysis underscore the potential for Reddit to offer an important space for self-disclosure for internet users who are experiencing pandemic-related mental health challenges. Integrating mental health supports into community-developed platforms that individuals are already using may therefore lead to increased uptake of these supports. Important to consider, however, is the potential unintended consequences of *how* these supports are integrated and received by online community members, particularly given previous research cautioning that online supports with referral to in-person treatments can inadvertently put some individuals at greater risk of active suicidal thinking (73). There is also a need for investigation and responsiveness to elsewhere-documented challenges of the widespread disinformation, hate, and extremism on this largely unmoderated platform, which impact Reddit's potential as a safe space for discussing personal challenges (74, 75). Additionally, Reddit is a public platform with posts visible and available to any viewer, raising concerns about its appropriateness as a mental health discussion and support forum. Although some users choose to use alternate and temporary usernames – termed ‘throwaway' accounts – to discuss personal topics (30), their posts and subsequent comments remain ‘public' and may be shared or reposted without consent and are subject to potentially malicious and unsupportive comments. As some posts in this analysis suggested that the content of particular subreddits perpetuated feelings of distress and anxiety, specific guidance for moderators and users of Reddit and subreddit communities on self-disclosure and ethical online engagement may be useful for the building of safer online spaces to meaningfully discuss mental health challenges. More broadly, further investigation is needed to explore these influences on online mental health self-disclosure while also identifying best practices and strategies for enhancing the relevance, acceptability, and safety of mental health supports provided in Reddit and other online spaces.

### Limitations

This paper is, to our knowledge, one of the first to qualitatively analyze pandemic-related mental health narratives on Reddit. However, there are important limitations to consider. Firstly, as our analysis drew from only one Reddit community, posts from other COVID-19 or mental health subreddits may present additional themes related to pandemic stressors and suicidal thoughts. Additionally, while various search terms were tested in advance of data collection, our search strategy may not have captured all posts expressing suicidal thoughts within *r/COVID19_support*; however, as this analysis sought qualitative themes across user posts, an exhaustive search was not considered necessary to provide insight into Reddit users' experiences. Examining demographic and geographic variation in users' experience may have added depth and further insight into study findings, but this was not possible because an inherent limitation of Reddit data is the absence of personal user details. The *r/COVID19_support* subreddit – and the broader Reddit platform – is a moderated space, with particular users designated as moderators with the ability to remove posts and delete comments (76). We are therefore unable to determine if posts discussing suicide during the pandemic were removed by moderators, or edited or deleted by original posters, prior to data collection. Further, *r/COVID19_support* is an English language subreddit (i.e., with community description, guidelines, and moderator posts in English); as our search term ‘suicide' is also English-language, study data did not include any non-English posts from Reddit users.

### Conclusions

Researchers have examined various drivers of suicidal thoughts in the context of the COVID-19 pandemic, and emerging quantitative data has begun to suggest associations between particular pandemic-related stressors and adverse mental health outcomes. In complement with this evolving work, this study extends understandings of the multiple and intersecting self-reported stressors contributing to suicidal thoughts in the context of the pandemic. Findings from this analysis demonstrate that some individuals are turning to Reddit as an online space to share experiences of suicidal thoughts and seek support in coping with the pandemic, suggesting potential for social media spaces to offer forums for discussing mental health challenges. However, posts illustrate users' serious suicidal thoughts, ongoing challenges with pandemic-related stressors, and a lack of effective strategies for managing poor mental health, echoing widespread global concerns of the significant mental health impacts of the COVID-19 pandemic.

## Data Availability Statement

The raw data supporting the conclusions of this article will be made available by the authors, without undue reservation.

## Ethics Statement

Ethical review and approval was not required for the study on human participants in accordance with the local legislation and institutional requirements. Written informed consent for participation was not required for this study in accordance with the national legislation and the institutional requirements. Written informed consent was not obtained from the individual(s) for the publication of any potentially identifiable images or data included in this article.

## Author Contributions

AS and EJ co-led conceptualization of the study, including study design. AS and CM contributed to data collection and analysis. AS led manuscript preparation. CM, TG, and EJ contributed to data interpretation and writing of the manuscript. LM and ES contributed to writing of the manuscript. All authors read and approved the final manuscript. EJ is the primary investigator and lead researcher on this study and associated studies assessing the mental health impacts of COVID-19. All authors are accountable for the content of the work.

## Conflict of Interest

The authors declare that the research was conducted in the absence of any commercial or financial relationships that could be construed as a potential conflict of interest.

## Publisher's Note

All claims expressed in this article are solely those of the authors and do not necessarily represent those of their affiliated organizations, or those of the publisher, the editors and the reviewers. Any product that may be evaluated in this article, or claim that may be made by its manufacturer, is not guaranteed or endorsed by the publisher.
